# Electrospinning Synthesis and Photocatalytic Activity of Mesoporous TiO_2_ Nanofibers

**DOI:** 10.1100/2012/154939

**Published:** 2012-04-24

**Authors:** Jing Li, Hui Qiao, Yuanzhi Du, Chen Chen, Xiaolin Li, Jing Cui, Dnt Kumar, Qufu Wei

**Affiliations:** Key laboratory of Eco-textiles, Jiangnan University, Ministry of Education, Wuxi 214122, China

## Abstract

Titanium dioxide (TiO_2_) nanofibers in the anatase structure were successfully prepared via electrospinning technique followed by calcination process. The morphologies, crystal structure, surface area, and the photocatalytic activity of resulting TiO_2_ nanofibers were characterized by field emission scanning electron microscopy (FE-SEM), transmission electron microscopy (TEM), X-ray diffraction (XRD), nitrogen sorption, and UV-vis spectroscopy. The results revealed that calcination temperature had greatly influenced the morphologies of TiO_2_ nanofibers, but no obvious effect was noticed on the crystal structure of TiO_2_ nanofibers. The photocatalytic properties of TiO_2_ nanofibers were evaluated by photocatalytic degradation of rhodamine B (RhB) in water under visible light irradiation. It was observed that TiO_2_ nanofibers obtained by calcination at 500°C for 3 hours exhibited the most excellent photocatalytic activity. We present a novel and simple method to fabricate TiO_2_ nanofibers with high-photocatalytic activity.

## 1. Introduction

In recent years, titanium dioxide (TiO_2_) has attracted much attention, which is because of its wide range of potential applications in environmental remediation, electronics, sensor technology, solar cell, and other related fields [1–6]. Among those applications, TiO_2_ has the most successful application in photocatalyst field due to its excellent photoactivity, high stability, and low cost.

A lot of research have been done on degradation industrial dye pollutants by using TiO_2_ as a photocatalyst. Especially, much effort has been devoted in modifying and improving photocatalytic activity of TiO_2_ [7–9]. Li et al. designed porous TiO_2_ nanofiber by alkali-disolution of SiO_2_ from TiO_2_/SiO_2_ composite nanofibers to enhance its surface-to-volume ratio, which improved the photocatalytic activity of TiO_2_ nanofiber without adding silica, and its best photocatalytic efficiency obtained in his experiment was 76.56% after 1 h irradiation [10]. Stengl et al. fabricated tungsten-doped titania by thermal hydrolysis of aqueous solutions of peroxo complexes of titanium and tungsten, which enhanced the reaction rates for photodegradation of Orange II dye [11]. Slimen et al. prepared TiO_2_/activated carbon composites by sol-gel technology, photocatalytic activity of which was greater than that of TiO_2_ Degussa P25 on the degradation of methylene blue in aqueous solution under visible irradiation [12]. Meng et al. fabricated TiO_2_ nanofibers with TiO_2_ nanorods growth on the surface, which exhibited better photocatalytic activity than pure TiO_2_ nanofibers [13]. Kuvarega et al. prepared nitrogen/palladium-codoped TiO_2_ by a modified sol-gel method to tune the electronic structure of TiO_2_ and improve its photocatalytic activity under visible light [14]. Kanjwal et al. prepared electrospun TiO_2_ nanofiber with silver nanoparticles and obtained strongly effective photocatalyst [15]. Gong et al. fabricated titanium oxide nanotubes by anodic oxidation of a pure titanium sheet in an HF solution, the as-prepared titanium oxide nanotubes had open structure at the top and closed structure at the bottom, with a controllable pore size ranging from 25 to 65 nm, which has very promising application in catalytic, biomedical areas [16]. Das et al. prepared TiO_2_ nanotubes by anodization process and studied the cell-material interaction, the results indicated that TiO_2_ nanotubes had better human osteoblast cell adhesion and spreading and provided much more excellent anchorage sites for filopodia extensions, when compared to polished Ti-control surface [17]. By all these above-mentioned techniques and methods, TiO_2_ with good photocatalytic activity could be prepared. However, some of all these methods involve some complicated processes, which would introduce complicated parameters into the preparation process of TiO_2_ photocatalyst, and some of the parameters would be difficult to control; some of all these methods involve comparatively higher production cost.

In this study, we successfully demonstrated a very simple way to fabricate TiO_2_ nanofibers by electrospinning in combination with calcination, but without combination with the conventional sol-gel technique. By adopting a novel method, production of TiO_2_ nanofibers in this way will optimize a lot of processing factors of the sol-gel method, which will affect the quality of the fabricated TiO_2_ nanofibers. The photocatalytic properties of TiO_2_ nanofibers prepared by our method were evaluated by photocatalytic degradation of rhodamine B (RhB) in aqueous solution under visible light irradiation, the photocatalytic activity of TiO_2_ nanofibers calcined at 500°C had the best photocatalytic activity and obtained 99% degradation rate under visible light irradiation for 2.5 h.

## 2. Experimental

### 2.1. Materials

 Poly (vinylpyrrolidone) (PVP; Mw = 1300000 g/mol), was purchased from Shanghai Qifuqing Material Technology Co., Ltd. Anhydrous ethanol (AR) and tetrabutyl titanate (TBT; CP) were purchased from Sinopharm Chemical Reagent Co., Ltd. All the materials were used as received, without further purification.

### 2.2. Preparation of TiO_2_ Nanofibers

 In a typical procedure, 0.01 mol TBT was added into 0.1 mol anhydrous ethanol in the capped bottle, followed by magnetic stirring for 5–10 min, to form a homogenous solution, then the calculated amount of PVP was dissolved into the solution, where the weight ratio of PVP and TBT was 1 : 9. The mixture was stirred by magnetic stirring until the formation of transparent and homogenous solution. The obtained solution was loaded into a 20 mL of a plastic syringe equipped with a 0.7 mm inner diameter of blunted stainless steel needle. The electrospinning setup utilized in this study was made in-house. The positive electrode of a high-voltage supply, which can generate DC voltage up to 50 kV, was connected to the needle, and its negative electrode was connected to the collecting panel, covered with an aluminum foil. The solution was delivered, via a syringe pump, to control the flow rate. The electrospinning parameters in this paper were set at a flow rate of 1.0 mL/h, the distance between the needle tip and the collecting panel of 24 cm and an applied positive voltage of 16 kV. The electrospun PVP/TBT composite nanofibers were deposited on the collecting panel to form fibrous mats.

 The obtained PVP/TBT composite nanofibers mats were calcined in air atmosphere for 3 hours at 500°C, 600°C, and 700°C, respectively, with a heating rate of 0.5°C/min.

### 2.3. Structural Characterization

 The morphologies of PVP/TBT composite nanofibers and TiO_2_ nanofibers were observed using a S4800 field emission scanning electron microscopy (FESEM). Transmission electron microscopy (TEM) was performed using a JEOL JEM-2100 Transmission electron microscopy instrument. X-ray powder diffraction patterns were obtained using D8 Advance X-ray diffractometer using Cu-K_*α*_ (*λ* = 1.5406 Å) irradiation over Bragg angles from 10 to 80°. The nitrogen absorption and desorption isotherms at 77 K were measured using Micrometrics Gemini V2.0 system after samples were vacuum-dried at 180°C overnight, and the surface area was calculated using the standard Brunauer-Emmett-Teller (BET) equation.

### 2.4. Photocatalytic Activity Test

 In order to investigate the photocatalytic activities of the samples, degradation of rhodamine (RhB) in an aqueous solution was performed using the prepared TiO_2_ nanofiber samples as photocatalyst under visible light irradiation. A 500-Watt tungsten halogen lamp was placed inside a cylindrical vessel and surrounded by a circulating water jacket to cool the lamp. An appropriate cutoff filter was chosen to make sure that the light wavelength permeated from the filter was no shorter than 420 nm, which ensured that the irradiation was in the visible light wavelengths only. The initial concentration of RhB was 5 mg/L. The amount of photocatalyst used was 0.1 g in a 100 mL aqueous solution of RhB. The solutions were continuously stirred, in dark for about 1.5 h, to obtain a good dispersion and establish adsorption-desorption equilibrium between RhB and photocatalyst, the solution was then illuminated under visible light, obtained from the tungsten halogen lamp. The distance, between the light source and the bottom of the solution, was about 15 cm, and the temperature of the solution stirred by dynamoelectric stirrer in an open reactor was about 25°C. At a given irradiation time intervals, the solution containing the prepared TiO_2_ nanofiber samples, was sampled (4 mL) each time and centrifuged for 15 min at a speed of 12000 rpm. The obtained dye filtrates were analyzed by a U-3310 UV-vis spectrometer (HITACHI), the absorption spectrum of each solution was measured over 200–700 nm. In the recorded spectrum, the absorbance reduction in the absorbance peak at *λ* ~ 552 nm was used to estimate the photodegradation efficiency and evaluate the photocatalytic activities of the TiO_2_ nanofiber samples.

## 3. Results and Discussion

### 3.1. XRD Patterns

**Figure 1:**
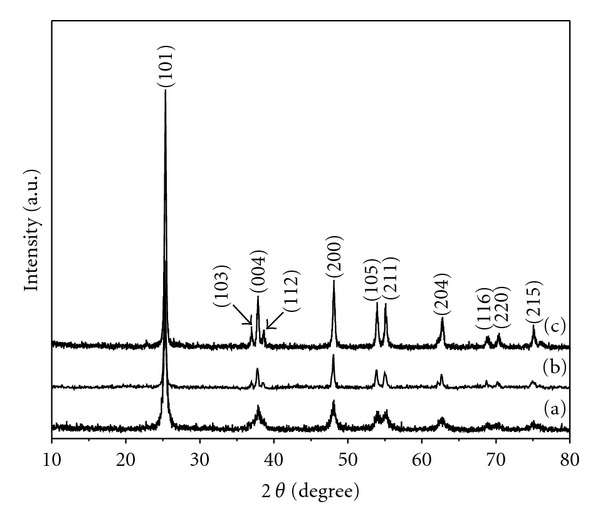
XRD patterns of the as-prepared samples calcined at (a) 500°C, (b) 600°C, and (c) 700°C.

Figure 1 shows that the XRD patterns of the as-prepared samples calcined at 500°C, 600°C, and 700°C for 3 hours in air atmosphere, respectively. The peaks shown in the XRD patterns correspond to the (101), (103), (004), (112), (200), (105), (211), (204), (116), (220), and (215) planes of TiO_2_ tetragonal anatase phase. These patterns can be well indexed to tetragonal anatase (JCPDS no. 21-1272, space group: I41/amd (141)). No peaks of brookite or rutile phase were detected, which indicate the high purity of the as-prepared samples. The crystallite sizes of the samples were calculated by Debye-Scherrer formula on the diffraction peaks of anatase (101) crystallite plane. According to the calculation results, the average crystallite sizes of the samples were 19.8, 31.8, and 33.0 nm, respectively. These results revealed that the resultant samples obtained at different calcination temperatures were all in the TiO_2_ anatase phase, and with an increase of the calcination temperature, grain size of the samples was getting larger.

### 3.2. SEM and TEM Images

**Figure 2:**
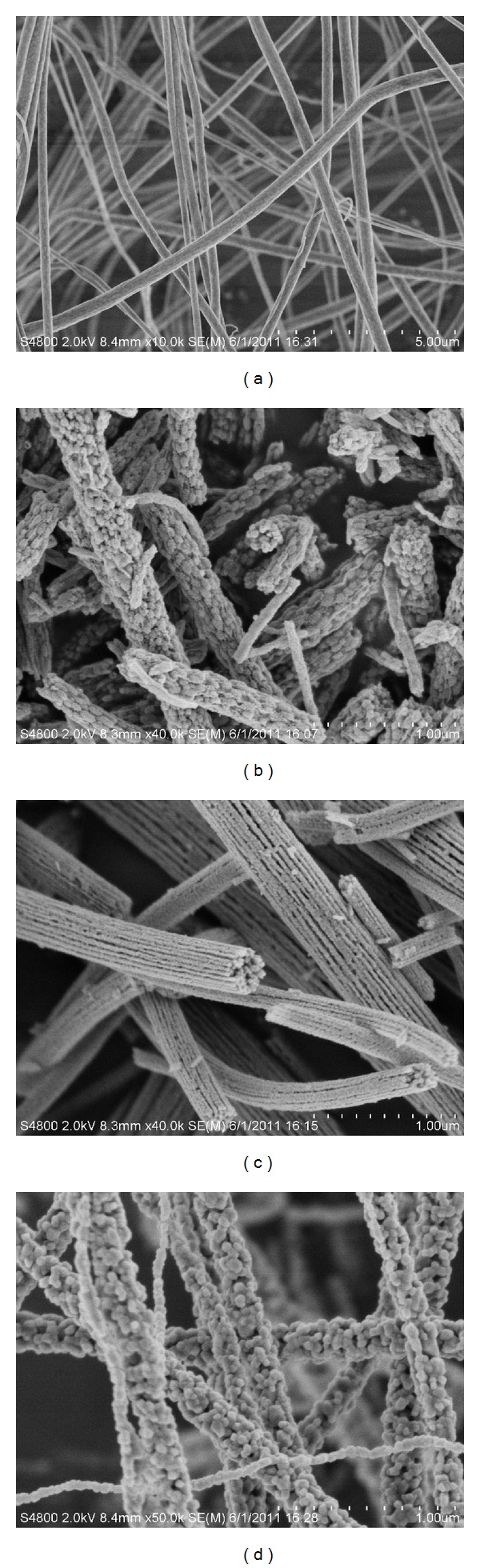
SEM images of the as-prepared (a) PVP/TBT composite nanofibers by electrospinning and thereof TiO_2_ nanofibers sintered at (b) 500°C, (c) 600°C, and (d) 700°C for 3 hours in air, respectively.

**Figure 3:**
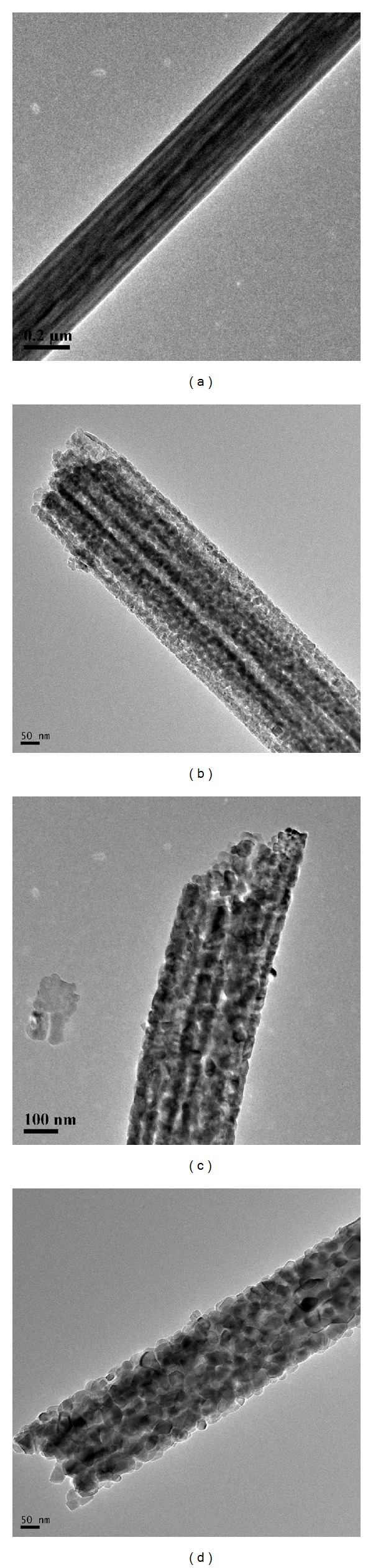
TEM images of (a) the PVP/TBT composite nanofibers and TiO_2_ nanofibers obtained at (b) 500°C, (c) 600°C and (d) 700°C, respectively.

The morphologies of the as-prepared PVP/TBT composite nanofibers and thereof TiO_2_ nanofibers are shown in Figure 2. It is clearly observed that the PVP/TBT composite nanofibers formed a fibrous structure with varying fiber diameters, as revealed in Figure 2(a). The electrospun PVP/TBT composite nanofibers showed smooth surface with fiber diameters, ranging from 100 to 450 nm. The calcinations significantly altered the surface morphologies of the electrospun nanofibers, as presented in Figures 2(b) and 2(d). It is evident that the diameters of the corresponding TiO_2_ nanofibers got smaller than the electrospun ones, after calcination process. The diameters of TiO_2_ nanofibers ranged from 70 to 350 nm for nanofibers obtained at 500°C, from 52 to 320 nm for nanofibers obtained at 600°C, and from 55 to 230 nm for nanofibers obtained at 700°C. It could also be found that different calcinations temperatures affected the morphologies of the obtained TiO_2_ nanofibers. TiO_2_ nanofibers obtained at 500°C and 700°C were composed of TiO_2_ nanoparticles, aggregated along fiber orientation, but TiO_2_ nanofibers obtained at 600°C were comprised of a bundle of nanofibrils that were aligned in the fiber orientation. The TEM images confirmed the SEM observations, as shown in Figure 3. As shown in Figure 3, it was obvious that TiO_2_ nanoparticles which composed of TiO_2_ nanofibers grew larger with the increase of calcination temperature, which was in accordance with XRD result, as shown in Figure 1. It also could be seen that TiO_2_ nanoparticles piled compactly along fiber orientation with the increase of calcination temperature from Figure 3.

### 3.3. Nitrogen Sorption

**Figure 4:**
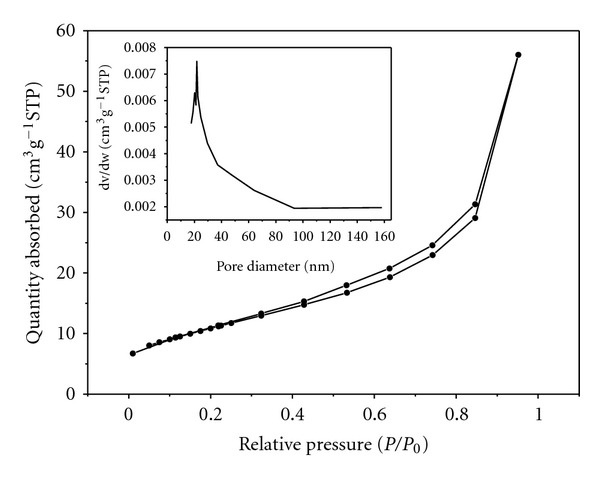
Nitrogen adsorption-desorption isotherm and pore size distribution curve (inset) of the as-prepared TiO_2_ nanofibers obtained at 600°C.

Figure 4 presents the nitrogen adsorption-desorption isotherm and Barrett-Joyner-Halenda (BJH) pore size distribution curve (inset) of the as-prepared TiO_2_ nanofibers obtained at 600°C. The isotherm belonged to type II according to IUPAC classification [18], which is a typical characteristic adsorption-desorption isotherm of mesoporous materials. These mesopores could be formed by the aggregation of TiO_2_ nanoparticles along the fiber orientation during calcination process. The quantity of nitrogen adsorption and desorption increased swiftly with increasing relative pressure in the range of 0.4 < *P*/*P*_0_ < 0.9, which revealed that the pore size distribution was quite narrow. The Brunauer-Emmett-Teller (BET) specific surface area of TiO_2_ nanofibers was 39.5 m^2^/g. As all the TiO_2_ nanoparticles obtained at 500°C, 600°C, and 700°C aggregated along the same direction in the same way, the diameter of the mesopores formed between aggregated TiO_2_ nanoparticles would depend on the size of TiO_2_ nanoparticles. As the grain size increased with the increase of calcination temperature, the specific surface area would decrease, which indicated that the specific surface area would be greater than that of samples obtained at 600°C, and 700°C.

### 3.4. Photocatalytic Activities

The structures of the dye molecules directly decide the absorption characteristic of dyes for light. In the electron absorption spectra of dyes, there are several absorption bands, which reflect the state of motion of the electrons. The absorption wavelength, absorption intensity, and the shape of absorption band are related directly to the structure of dye molecules. Therefore, it is possible to evaluate the structural variation of dyes by investigating the variation of the electron absorption spectra during the process of degradation of the dyes.

It has been reported that the photodecomposition of RhB aqueous solution in the presence of TiO_2_ particles as a photocatalyst has two pathways: (1) the photocatalytic pathway, which would occur under UV irradiation. In this pathway, TiO_2_ would be activated to generate electrons under UV irradiation (*λ* ≤ 385 nm) to drive the process of photodegradation; (2) the photosensitization pathway, which usually occurs under visible light irradiation. The band gap of anatase TiO_2_ is 3.2 eV; therefore, the energy of visible light (*λ* > 400 nm) is not enough to excite TiO_2_ to produce the electron to drive the process of photodegradation. In the photosensitization pathway, where TiO_2_ cannot be activated by visible light, dyes will absorb visible light irradiation and can be excited, which will drive the process of photodegradation, but the existence of TiO_2_ photocatalyst is a prerequisite and a crucial requirement to ensure electron carriers to electron acceptors adsorbed on the TiO_2_ surface, which will help in the process of photodecomposition [17, 18]. In our paper, the photodegradation of RhB as a target pollutant in water media was performed under visible light (*λ* > 400 nm) in the presence of TiO_2_ nanofibers obtained in this paper as a photocatalyst. Therefore, the photosensitization pathway would dominate the process of photodegradation of RhB. 

**Figure 5:**
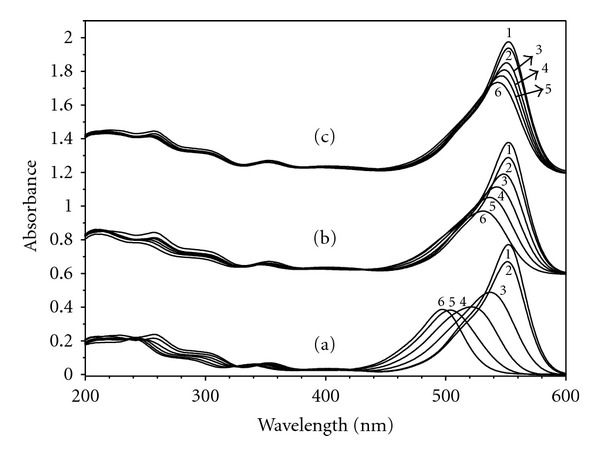
UV-vis spectral changes of RhB aqueous solution (5 mg/L) at different irradiation times; spectra 2, 3, 4, 5, and 6 denote different irradiation times of 0, 1, 1.5, 2.0, and 2.5 hours, respectively. The spectrum 1 is the UV-vis spectrum of RhB aqueous solution before the addition of TiO_2_ nanofibers as photocatalyst. Three sets of spectra (a), (b), and (c) belong to TiO_2_ nanofibers obtained at 500°C, 600°C, and 700°C, respectively.

**Figure 6:**
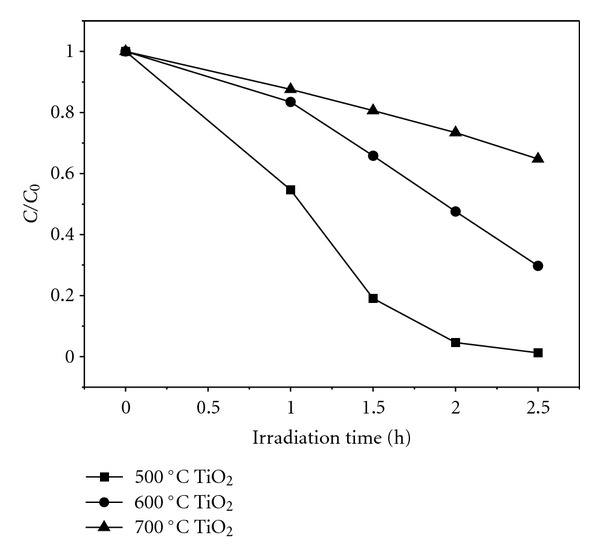
Photodegradation rate of RhB solution in the presence of the as-prepared TiO_2_ nanofibers obtained at 500°C (solid square), 600°C (solid circle), and 700°C (solid triangle) under visible light.

RhB was used for evaluating the photocatalytic activity of the obtained TiO_2_ nanofibers in this paper. The temporal photosensitized transformation of RhB in RhB/500°C-TiO_2_, RhB/600°C-TiO_2_, and RhB/700°C-TiO_2_ solution systems and their corresponding wavelength shifts in the major absorption band were recorded in Figure 5. The photodegradation rate of RhB in the presence of the as-prepared TiO_2_ nanofibers obtained at 500°C, 600°C, and 700°C is shown in Figure 6. It was revealed that 99%, 70% and 35% degradation of RhB were recorded after 2.5 hours of irradiation of the samples of 500°C-TiO_2_, 600°C-TiO_2_ and 700°C-TiO_2_ nanofibers, respectively, as shown in Figure 6. It was also obviously observed that TiO_2_ nanofibers obtained at 500°C have exhibited the best photocatalytic activity among the samples tested. Moreover, it could be easily found that the major absorption band of RhB in RhB/500°C-TiO_2_ solution system had the greatest wavelength shift (hypsochromic shifts), where in RhB/600°C-TiO_2_ solution system smaller hypsochromic shifts were observed, and in RhB/700°C-TiO_2_ solution system it had the slightest wavelength shift, as presented in Figure 5. These results were in accordance with the degradation rate of the as-prepared TiO_2_ nanofibers shown in Figure 6. It has been reported that the wavelength shift was caused by deethylation of RhB [19, 20].

In our current paper, it was suggested that all the samples exhibited very good crystal structure and the same anatase phase based on the XRD analysis, but the difference in the degradation rate was very much remarkable, which indicated that the crystal phase in here had no significant effect on the degradation. By examination of the spectra in Figure 5, especially (a) spectra in Figure 5, it suggests that RhB was deethylated in a stepwise way (ethyl groups were removed one by one as confirmed by the gradual shifts of hypsochromic shifts). Deethylation of the N,N,N′,N′-tetraethylated rhodamine molecules (RhB) had the wavelength position of its major absorption band moved toward the blue region, *λ*_max⁡_, RhB, 552 nm; N,N,N′-triethylated rhodamine, 539 nm; N,N′-diethylated rhodamine, 522 nm; N-ethylated rhodamine, 510 nm; and rhodamine, 498 nm [20]. The data shown in (a) spectra of Figure 5 were almost identical with the data presented in [20], which suggested that TiO_2_ nanofibers obtained at 500°C in this paper decomposed RhB because of photosensitization of RhB. Among all the samples, TiO_2_ nanofibers obtained at 500°C aided the self-photosensitization of RhB very well, and the other two samples did not work well with RhB for degradation.

Many reports showed that the photocatalytic activity of TiO_2_ was obviously influenced by many factors, such as grain size, crystallization, morphology, and specific surface area [21, 22]. However, none of these factors are not decisive factor to the photocatalytic activity of TiO_2_, only these factors could work in coordination with each other to reach a great synergistic effect, TiO_2_ could achieve great photocatalytic activity. TiO_2_ nanofibers obtained at 500°C had the most excellent photocatalytic activity among all the as-prepared samples, the reasons are as follows. TiO_2_ nanoparticles composed of TiO_2_ nanofibers obtained at 500°C had the smallest grain size, and its crystallinity was also pretty well, though lower than that of samples prepared at 600°C, and 700°C, as XRD results shown. TiO_2_ nanofibers obtained at 500°C would have greater specific surface area, as discussed in nitrogen sorption section, which would enhance the adsorption of dyes around/on the surface of TiO_2_ nanoparticles, that means more photogenerated electrons would be created, resulting in high photodegradation rate. Those factors of TiO_2_ nanofibers obtained at 500°C were all favored its photocatalytic activity, therefore, the highest photocatalytic activity of TiO_2_ nanofibers obtained at 500°C could be attributed to the results of the synergistic effects of grain size, crystallization, morphology, and specific surface area. Though TiO_2_ nanofibers obtained at 600°C, and 700°C had the similar morphology with that of as-prepared nanofibers at 500°C, their grain size were much larger, and their specific surface area were lower than that of nanofibers obtained at 500°C.

## 4. Conclusions

In our current paper, mesoporous TiO_2_ nanofibers with anatase phase were successfully prepared by electrospinning in combination with calcination processwithout using the conventional sol-gel technique. The TiO_2_ nanofibers obtained at 500°C had the best photocatalytic activity among all the samples, and it aided the self-photosensitization of RhB well to achieve 99% degradation rate. This study revealed that TiO_2_ nanofibers obtained by our promising novel method could be a very basis for photocatalysis by using the solar light.
